# The pursuit of therapy for progeria

**DOI:** 10.18632/aging.203254

**Published:** 2021-06-27

**Authors:** Saurabh Saxena, Dhananjay Shukla

**Affiliations:** 1 NextGen In Vitro Diagnostics Pvt. Ltd, BSC-BioNest Bioincubator, Regional Centre for Biotechnology, NCR-Biotech Science Cluster, Faridabad, Haryana 21001, India; 2 Department of Biotechnology, Guru Ghasidas Vishwavidyalaya, Bilaspur, Chhattisgarh 495009, India

**Keywords:** aging, Hutchinson-Gilford Progeria Syndrome, therapeutics

Since the discovery of the point mutation responsible for Hutchinson-Gilford Progeria Syndrome (HGPS) in 2003, notable achievements have been made in the development of its therapy. The first drug against HGPS and progeroid syndromes, lonafarnib, has now received approval from the US Food & Drug Administration (FDA) in November 2020 to administer in patients of age one year and above with a body surface area of ≥ 0.39 m^2^ [1]. It is an orally active compound that works as a farnesyltransferase inhibitor (FTI), preventing farnesylation and accumulation of progerin at the site of the nuclear lamina. Lonafarnib is sold as Zokinvy^TM^ by Eiger BioPharmaceuticals with a license from Merck & Co. Inc. Its administration has been found to increase the life span and reduce the chances of stroke in HGPS patients [2]. A phase 1/2 dose-escalation clinical trial on everolimus combined with lonafarnib is in process (NCT02579044).

There are several other therapeutic approaches under investigation for the treatment of HGPS. They include inhibition of post-translational modifications of progerin, regulation of oxidative stress and mitochondrial dysfunction, control of DNA damage, epigenetic modifications, management of inflammatory pathways of the cell, reestablish the nuclear transport, removal of progerin by autophagy, suppression of progerin-lamin A binding, dietary modifications, supplementation with fecal microbes of healthy peers, pharmacological elimination of aging cells, etc. Apart from these, there are few approaches that can actually target the causative mutation (c.1824 C > T; p.G608G) or the mRNA of defective lamin A protein *viz.* CRISPR-Cas9 system, suppression of aberrant splicing, and the use of therapeutic RNA [3]. However, because HGPS is a systemic disorder that affects most of the types of cells in the body, the treatment of all the affected cells with any therapeutic approach is a challenging task. In our previous paper [3], we had emphasized the evaluation of extracellular vesicles (EVs) or exosomes derived from healthy stem cells for the treatment of HGPS. EVs from healthy cells contain many bioactive factors that may restore the normal functioning of a cell. They have already been found effective in increasing the life span of various cellular and animal models of premature aging. Bioengineered EVs can also be loaded with therapeutic molecules to enhance their delivery efficiency at the systemic level.

Barcena et al. [4] have even linked aging with the gut microbiome, suggesting an abundance of phylum Verrucomicrobia bacteria and reduced Proteobacteria population in long-lived humans/mice; but vice-versa in progeric ones. They demonstrated an extended (13.5%) life span in mouse model of HGPS (*Lmna^G609G/G609G^*) after the oral administration of fecal microbes originated from healthy/wild-type peers. Similarly, transplantation with Verrucomicrobia *Akkermansia muciniphila* bacteria alone could prolong the lifetime in the same mouse model. However, the efficacy of the amelioration of aging-associated dysbiosis on life span in HGPS patients is needed to be evaluated.

A unique approach called ‘*in vivo* base editing’ has been developed recently that may correct the LMNA mutation responsible for progerin production and accumulation [5]. In this study, Koblan et al. introduced an adenine base editor (ABE) that converts A•T base pair into G•C, coupled with sgRNA into the mouse model of HGPS. Adeno-associated virus 9 (AAV9) containing ABE cassette was injected via retro-orbital injection at a high dose of 10^11^ or 10^12^ viral genomes (vg) per animal into mice aged 3 or 14 days. It was selected as a delivery vehicle because of its higher delivery efficiency towards the major progeria-related tissues like the heart and muscles. The ABE-treated mice exhibited a 1.8-fold to 2.4-fold increase in life span with a significant improvement in vascular pathology and subcutaneous body fat as compared to controls. At the cellular level, it corrected HGPS mutation, reduced progerin expression level, and ameliorated nuclear morphology in progeric mice. The editing efficiencies in different organs after 6 months of treatment varied from 20% to 60%. There are certain advantages of base editing over genetic editing like higher efficiency and precision without the requirement of a donor DNA template and double-strand breakage (DSB). The initial results in mice are encouraging; however, more optimization of the technique, in terms of delivery efficiency and immunogenicity towards the capsid of AAV and/or ABE cassette is required before proceeding to clinical trials.

In another important development, a molecule called Progerinin (or SLC-D011) has been synthesized that can interfere with the progerin-lamin A binding [6]. It was found that daily oral delivery of Progerinin (50 mg/Kg body weight) to *Lmna^G609G/G609G^* and *Lmna^G609G/+^* mice can increase the life span for around 10 and 14 weeks respectively. It looks very promising as Progerinin was found more effective than lonafarnib, which could prolong the life span of *Lmna^G609G/+^* mice up to 2 weeks only. Progerin administration also ameliorated progeroid phenotype, thus improving the quality of life in these mice. A Phase I clinical trial in healthy volunteers is already proceeding to evaluate the safety, tolerability, pharmacokinetics, and pharmacodynamic profile of Progerinin after single and multiple doses (NCT04512963).

Overall, the present therapeutic strategies for HGPS that are under evaluation can be broadly divided into two categories: (i) those affecting progerin production, modification, clearance, and functioning (ii) those managing the downstream effects of progerin accumulation at molecular or physiological level. For an effective remedy of HGPS, the biomolecular pathways of both the categories must be encountered. Moreover, advancements in the techniques like base editing and CRISPR/Cas-9 system gene editing can be really helpful for the treatment of genetic disorders including HGPS.
